# Genetic diversity and population structure analysis of spinach by single-nucleotide polymorphisms identified through genotyping-by-sequencing

**DOI:** 10.1371/journal.pone.0188745

**Published:** 2017-11-30

**Authors:** Ainong Shi, Jun Qin, Beiquan Mou, James Correll, Yuejin Weng, David Brenner, Chunda Feng, Dennis Motes, Wei Yang, Lingdi Dong, Gehendra Bhattarai, Waltram Ravelombola

**Affiliations:** 1 Department of Horticulture, University of Arkansas, Fayetteville, AR, United States of America; 2 Crop Improvement and Protection Research Unit, US Department of Agriculture, Agricultural Research Service (USDA-ARS), Salinas, CA, United States of America; 3 Department of Plant Pathology, University of Arkansas, Fayetteville, AR, United States of America; 4 Dep. of Agronomy, Plant Introduction Station, G212, Agronomy Hall, Iowa State Univ., Ames, IA, United States of America; 5 Vegetable Research Center, University of Arkansas, Alma, AR, United States of America; Washington University, UNITED STATES

## Abstract

Spinach (*Spinacia oleracea* L., 2n = 2x = 12) is an economically important vegetable crop worldwide and one of the healthiest vegetables due to its high concentrations of nutrients and minerals. The objective of this research was to conduct genetic diversity and population structure analysis of a collection of world-wide spinach genotypes using single nucleotide polymorphisms (SNPs) markers. Genotyping by sequencing (GBS) was used to discover SNPs in spinach genotypes. Three sets of spinach genotypes were used: 1) 268 USDA GRIN spinach germplasm accessions originally collected from 30 countries; 2) 45 commercial spinach F1 hybrids from three countries; and 3) 30 US Arkansas spinach cultivars/breeding lines. The results from this study indicated that there was genetic diversity among the 343 spinach genotypes tested. Furthermore, the genetic background in improved commercial F1 hybrids and in Arkansas cultivars/lines had a different structured populations from the USDA germplasm. In addition, the genetic diversity and population structures were associated with geographic origin and germplasm from the US Arkansas breeding program had a unique genetic background. These data could provide genetic diversity information and the molecular markers for selecting parents in spinach breeding programs.

## Introduction

Spinach (*Spinacia oleracea* L.) has become an increasingly important economic vegetable crop worldwide with an estimated annual value of $11.8 billion (Correll et al. 2011; van Deynze 2014) [1, 2]. The US is the second largest producer of spinach after China with over 550,000 tons of spinach harvested, valued at over $300 million annually (Correll et al. 2011; NASS 2015) [1, 3]. In addition to its economic importance, spinach is one of the healthiest vegetables in the human diet due to its high concentration of nutrients and health-promoting compounds (Dicoteau 2000; Morelock 2005) [4, 5]. During the last 15 years, the US spinach industry has seen a dramatic increase in fresh market demand [3] (NASS 2015). This requires the development of improved cultivars to increase spinach production.

The success of a plant breeding program is largely dependent on the availability of genetically diverse plant germplasm to allow for cultivar improvement. The genetic diversity and population structure of a plant species allows geneticists and plant breeders to use the resources for crop improvement. Such an approach has benefited many crops, for example cucumber [6] (Ly et al. 2012), maize (Zhang et al. 2016) [7], rice (Kuwahara et al. 2014) [8], and soybean (Li et al. 2008) [9]. Genetic diversity and population structure also have been examined in spinach (Khattak et al. 2007) [10] examined the genetic diversity of 33 spinach hybrid cultivars, from seven different breeding stations all over the world, using 13 SSR markers and the results showed that the spinach hybrids were grouped into three clusters; the first two of the three clusters consisted of European spinach types, which were well discriminated according to their origin from different breeding stations; the third cluster was a mixture of Asian as well as European types of spinach; and the subclusters in the third group did not reflect differences in morphology, earliness or company origin. Hu et al. (2007) [11] analyzed the genetic diversity among 38 USDA spinach germplasm accessions and 10 commercial hybrids of spinach using target region amplification polymorphism (TRAP) markers and found the average pair-wise genetic similarity coefficient (Dice) was 57.5% with a range from 23.2 to 85.3% and indicated that the genetic relationships among the accessions examined were not highly associated with the geographic locations in which the germplasm was collected. Eftekhari et al. (2010) [12] examined genetic diversity among 44 Iranian spinach landraces using 21 morphological characteristics under open field conditions and the materials were clustered into four groups containing 14, 4, 19 and 7 landraces, respectively: the first group included landraces with a similarity in leaf shape and female plant percent; in the second group, included materials with a similar leaf number and shape, pedicle length, growing period and fresh weight; the third group included landraces with spiny seed similar plant fresh weight and growing period; and the forth group included lines with a similar plant dry weight. Kuwahara et al. (2014) [8] assessed the genetic diversity among 50 spinach germplasm accessions collected from geographically diverse regions of West Asia, East Asia, Japan, Europe and the USA using SSR markers and found the genetic diversity was affected by geographical regions. Wu et al. (2013) [13] determined the genetic diversity among 110 spinach germplasms accessions collected from different geographical origins and identified two major groups, where group 1 was comprised of spinach which originated in European, America, West Asia, East Asia, and Northern China, whereas group 2 consisted of spinach originated in Southern China and Japan. The results suggested that Northern and Southern Chinese spinach populations may have different origins. Sabaghnia et al. (2014) [14] examined 54 spinach landraces collected from diverse geographical regions of Iran and divided the landraces into sixteen clusters based on for several qualitative and quantitative trait data. Ebadi-Segheloo et al. (2014) [15] evaluated 121 spinach landraces, collected from the various spinach growing areas of Iran, using several agro-morphological traits such as leaf area, leaf width, petiole length, petiole diameter, seed yield and 1000-seed weight and identified six clusters with each cluster having some specific unique characteristics. Recently, Xu et al (2017) [16] analyzed the genetic diversity among 120 cultivated and wild spinach accessions including 107 cultivated *S*. *oleracea* and 13 wild accessions (5 *S*. *tetrandra* and 8 *S*. *turkestanica*) based on the transcriptome sequencing data and found that the 120 spinach accessions were clustered into three major groups: the first group consisted of *S*. *turkestanica* and *S*. *tetrandra* accessions; the second group contained cultivars from East Asia, Chinese commercial varieties and two cultivars from Pakistan and Russia; and the third group included cultivars from Central/West Asia, Europe, North America and Africa, as well as the remaining commercial cultivars.

With improved next generation sequencing (NGS), and the decreasing cost of sequencing, it is now feasible to discover millions of single-nucleotide-polymorphisms (SNPs) for any plant and connect these markers to desirable phenotypic traits. Spinach is a very popular vegetable crop which can greatly benefit from the development of molecular tools to improve commercial cultivars. Until recently, most of the breeding efforts to develop spinach cultivars against biotic and abiotic stresses have relied on conventional breeding approaches. Although this approach has yielded cultivars with improved characteristics, conventional breeding can be time-consuming, labor intensive, and expensive. Alternatively, spinach breeding can be accelerated by the utilization of molecular tools that can reduce the time and cost of screening plants for desired characteristics. Molecular plant breeding has been the foundation for 21st century crop improvement (reviewed by Moose and Mumm, 2008) [17]. Marker assisted selection (MAS) has been used successfully in selection of specific genes/alleles in crop improvement (Collard et al. 2005; Collard and Mackill 2008; Xu and Crouch 2008) [18–20]. DNA sequencing by NGS technologies (van Dijk et al. 2014) [21] using genotyping-by-sequencing (GBS) (Elshire et al. 2011) [22] can be applied to a wide array of organisms, including plants, for genome sequencing and SNP discovery. GBS is one of the genotyping approach using next-generation sequencing platforms that utilizes a simple highly-multiplexed system for constructing reduced representation libraries which reduces sample handling, requires fewer PCR and purification steps, no size fractionation and uses inexpensive barcoding (Elshire et al. 2011) [22]. GBS is also a rapid and less expensive approach for trait mapping and association, and can be used in molecular breeding and allow plant breeders to conduct genomic selection on any germplasm or species with and without prior knowledge of the genome (Elshire et al., 2011; Sonah et al., 2013) [22, 23] and GBS have been widely used for SNP discovery in trait mapping and is an inexpensive and fast approach (Elmer et al. 2015; Nimmakayala et al. 2014) [24, 25]. Thus, using a GBS platform can be powerful approach for genome-wide SNP discovery, genetic diversity analysis, genetic map construction, linkage mapping, genome-wide association, and MAS in spinach. We used GBS for SNP discovery in spinach in this study. The spinach genome Spinach-1.0.3 is available to the public at http://www.ncbi.nlm.nih.gov/Traces/wgs/?val=AYZV02 and also at “The Beta vulgaris Resource” web site with the page at http://bvseq.molgen.mpg.de/Genome/Download/Spinach/, representing approximately half of the spinach genome (Dohm et al. 2014; Minoche et al. 2015) [26, 27].

We used the AYZV02 as the reference of spinach genome sequences for short reads alignment and SNP discovery in each spinach sample in this study. The objective of this research was to conduct genetic diversity and population structure analyses in a collection of world-wide spinach genotypes using SNP markers.

## Materials and methods

### Plant materials and genetic diversity panels

A total of 343 spinach genotypes, including 268 accessions from the USDA-GRIN spinach germplasm collection, 45 commercial F1 hybrids, and 30 Arkansas spinach lines, were examined for spinach population structure and genetic diversity (Table 1, S1, S2 and S3 Tables). The 268 USDA-GRIN spinach accessions were originally collected from 30 countries, with a majority (82.5%) from ten countries: Turkey (n = 81), United States (US) (n = 46), China (n = 20), Macedonia (n = 16), Afghanistan (n = 15), Iran (n = 15), Belgium (n = 9), Hungary (n = 6), India (n = 6), and Japan (n = 6) (S2 and S4 Tables). Seeds of all 268 accessions were kindly provided by the USDA-ARS North Central Regional Plant Introduction Station at Iowa State University, Ames, IA. The 45 hybrids primarily from the Netherlands (n = 32) and the US (n = 5) (S3 Table). Among the additional eight hybrids, one was from France; three from American Takii Inc, which may have both US and Japan origins; and four F1 hybrids, Denali.F1, Hector.F1, Indian.Summer.F1, and Spinner.F1 from Johnny Selected Seeds without seed source information but possibly from the US (S3 Table). The seed of all 45 hybrids were kindly provide by the seed companies (S3 Table). The seed of all 30 Arkansas spinach lines was developed at the University of Arkansas (S1 Table).

**Table 1:** The information of 343 spinach genotypes used in this study including their original region, country, and seed sources.

**USDA-Grin** **Cowpea germplasm**	**Region**	**Seed source**	**Country**	**No. of** **Line**	**No. of** **Country**
	Asia	USDA-Grin	Afghanistan, China, Egypt, India, Iran, Iraq, Japan, Mongolia, Nepal, South Korea, Syria	74	11
	America	USDA-Grin	UnitedStates	46	1
	Europe_1	USDA-Grin	Belgium, Denmark, England, France, Germany, Greece, Hungary, Italy, Macedonia, Netherlands, Poland, Republic Georgia, Soviet Union, Spain, Thailand, United Kingdom	55	16
	Turkey	USDA-Grin	Turkey	81	1
	Africa	USDA-Grin	Ethiopia	1	1
	International. cultivar	USDA-Grin	unknown	11	
	sub-Total			268	30
**Arkansas** **cowpea**	US.Arkansas	Arkansas	US Arkansas	30	1
**F1 hybrids**	**Region**	**F1 hybrid**	**Country**	**No. of** **Line**	**No. of** **Country**
	Asia.America	F1	Japan/United State	3	?
	Europe	F1	France, Netherlands	33	2
	America?	F1	United States?	4	?
	America	F1	United States	5	1
	Sub-Total			45	3
Total				343	3

Based on the different resource and geographic origin of the spinach genotypes, the genetic diversity and population structure were analyzed separately by (1) the worldwide germplasm accessions of USDA-GRIN collection, (2) the commercial F1 hybrids, (3) by regions (groups), (4) by countries, (5) United States, (6) the Arkansas spinach lines, and (7) finally, combined all tested 343 spinach genotypes.

### DNA extraction, GBS, and SNP discovery

Genomic DNA was extracted from leaves of spinach plants using the CTAB (hexadecyltrimethyl ammonium bromide) method [28] (Hulbert and Bennetzen 1991). A DNA library was prepared using the restriction enzyme ApeKI following the GBS protocol described by Elshire et al. (2011) [22] and DNA sequencing was performed using GBS method [22, 23] (Elshire et al. 2011; Sonah et al. 2013). The 90 bp, double-end sequencing was performed on each spinach genotype (accession/F1 hybrid/cultivar/line) using the GBS protocol by an Illumina HiSeq 2000 machine at the Beijing Genomics Institute (BGI) in Hong Kong. GBS data alignment, mapping and SNP discovery were done using SOAP family software (http://soap.genomics.org.cn/) by the bioinformatics team at BGI. The GBS data provided by BGI averaged 3.26 M with 90 bp, short-read nucleotides for each spinach sample. The short reads of the GBS data were first aligned to the Spinach-1.0.3 spinach genome reference (AYZV02, http://www.ncbi.nlm.nih.gov/Traces/wgs/?val=AYZV02). The SOAP2 / SOAPsnp were used for SNP calling [29, 30] (Li, 2009; Luo et al., 2012). Approximately a half-million SNPs were discovered from the GBS data among the 343 spinach genotypes (germplasm accessions/F1 hybrids/lines), as provided by BGI. The spinach genotypes and SNPs were filtered before conducting genetic diversity analyses. If a spinach genotype had >20% missing SNP data and the heterozygous SNP genotype was >30%, the spinach genotype was removed from the panel. The SNP data were filtered to keep for minor allele frequency (MLF) >2%, missing data <7%, and heterozygous genotype <25%.

### Population structure and genetic diversity

The model-based program STUCTURE 2.3.4 [31] (Pritchard et al., 2000) was used to assess the population structure of each spinach set based on SNP loci postulated from GBS. In order to identify the number of populations (K) making up the structure of the data, the burn-in period was set at 10,000 with the Markov Chain Monte Carlo iterations and the run length set at 20,000 in an admixture model. The analysis then correlated allele frequencies independently for each run [6] (Lv et al., 2012). Ten runs were performed for each simulated value of K, which ranged from 1 to 10. For each simulated K, the statistical value delta K was calculated using the formula described by Evanno et al. (2005) [32]. The optimal K was determined using Structure Harvester [33] (Earl and von Holdt, 2012; http://taylor0.biology.ucla.edu/structureHarvester/). Each spinach genotype was then assigned to a cluster (Q) based on the probability determined by the software that the genotype belonged in the cluster. The cut-off probability for assignment to a cluster was 0.525 for only two clusters (structure populations), or 0.50 for three or more clusters. Based on the optimum K, a Bar plot with ‘Sort by Q’ was obtained to show the population structure among the spinach genotypes (accessions/hybrids/lines).

Genetic diversity also was assessed, and the phylogeny trees were drawn using MEGA 6 [34] (Tamura et al., 2013) based on the Maximum Likelihood tree method with the following parameters [35] (Shi et al. 2016) 2013). Test of Phylogeny: None, Model/Method: General Time Reversible model, Rates among Sites: Gamma distributed with Invariant sites (G + I), Number of Discrete Gamma Categories: 5, Gaps/Missing Data Treatment: Use all sites, ML Heuristic Method: Subtree-Pruning-Regrafting-Extensive (SPR level 5), Initial Tree for ML: Make initial tree automatically (Neighbor Joining), and Branch Swap Filter: Moderate. During the drawing of the phylogeny trees, the population structure and the cluster information were imported to MEGA 6 for combined analysis of genetic diversity. For sub-tree of each Q (cluster), the shape of ‘Node/Subtree Marker’ and the ‘Branch Line’ was drawn with the same color as in the figure of the Bar plot of the population clusters from the STRUCTURE analysis.

## Results

### Population structure and genetic diversity in the worldwide germplasm accessions of USDA-GRIN collection

**Fig 1:**
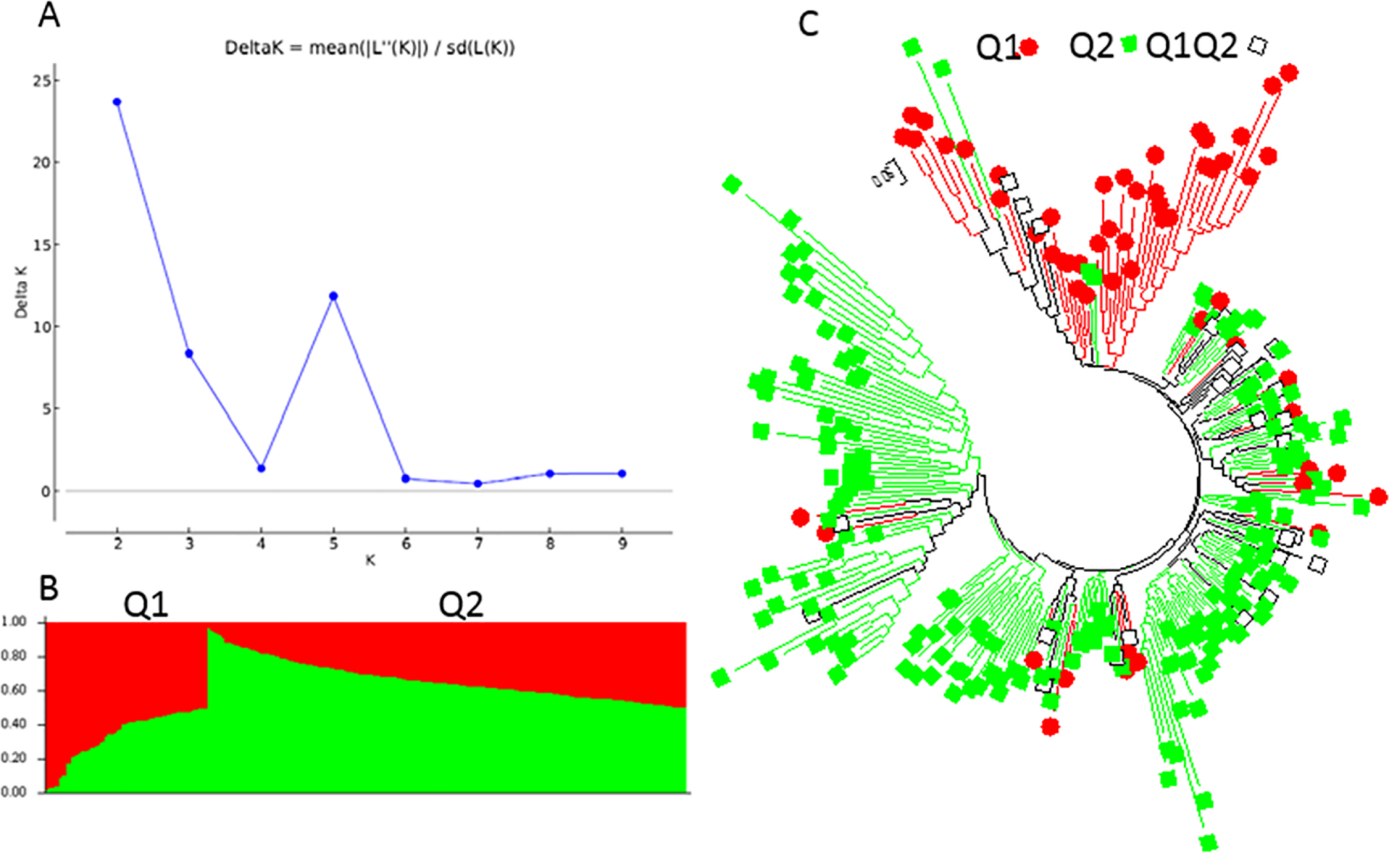
Model-based populations in association panels consisted of 268 USDA GRIN germplasm accessions: (a) Delta K values for different numbers of populations (K) assumed in analysis completed with the STRUCTURE software. (b) Classification of 268 spinach accessions into two populations using STRUCTURE Version 2.3.4, where the numbers on the y-axis show the subgroup membership, and the x-axis shows the different accession. The distribution of accessions into different populations is indicated by the color coding and shape (Cluster 1, Q1, is red round shape; and Cluster 2, Q2, is green squared). (c) Maximum Likelihood (ML) tree of the 268 spinach accessions drawn in MEGA 6. The color code for each population is consistent in the (b) and (c), and the empty black square represents accessions aligned with the admixture cluster or population, Q1Q2 in 268 USDA GRIN spinach germplasm accessions.

The population structure of the 268 USDA-GRIN germplasm accessions was initially inferred using STRUCTURE 2.3.4 [31] (Pritchard et al., 2000). The peak delta K was observed at K = 2, indicating the presence of two main population clusters, Q1 and Q2 in the spinach germplasm panel (Fig 1A and 1B). The classification of accessions into populations or clusters based on the model-based structure from STRUCTURE 2.3.4 is shown in Fig 1B and S2 Table. In total, 240 of 268 germplasm accessions (89.6%) were assigned to one of the two populations or clusters. Q1 and Q2 consisted of 57 (21.3%) and 183 (68.3%) accessions, respectively, and the remaining 28 accessions (10.4%) were categorized as having admixed ancestry Q1Q2 between Q1 and Q2 (S2 Table).

Based on the two structured populations or clusters Q1 and Q2 among the 268 spinach germplasm, the Q1 mainly consisted of the germplasm accessions from Asia (66.7% in Q1) and the Q2 from Europe (64.5% in Q2) (Table 2). Among the 74 Asia accessions, Q1, Q2, and admixed (Q1Q2) had 38, 22, and 14 accessions consisted of 51.4%, 29.7%, and 18.9%, respectively (Table 2). Among the 136 Europe accessions, Q1, Q2, and admixed (Q1Q2) had 8, 118, and 10 accessions consisted of 5.9%, 86.8%, and 7.4%, respectively (Table 2), indicating the majority of Europe accessions belonged to Q2. Among the 46 America accessions, Q1, Q2, and admixed (Q1Q2) had 11, 31, and 4 accessions consisted of 23.9%, 67.4%, and 8.7%, respectively (Table 2), indicating the majority of America accessions belonged to Q2. Among the 268 germplasm accessions, only one accession was from Africa and was clustered into Q2 and the 11 international cultivars without nationality information were grouped into Q2 as well (Table 2).

**Table 2:** Three parameters were estimated: (1) The number of spinach accessions in each cluster was list by region (up section in the table); (2) the percentage of accessions in each cluster was listed by region and the data were shown in each row (the middle section in the table); and (3) the percentage of accessions in each region was listed by cluster and the data were shown in each column (bottom section in the table) in the 268 USDA spinach germplasm accessions.

Region	No. of accessions in each cluster by region										
	Based on two clusters				Based on five clusters						
	Q1	Q2	Admixture	sum	q1	q2	q3	q4	q5	Admixture	sum
Asia	38	22	14	74	14	1	5	15	37	2	74
America	11	31	4	46		2	28	9	7		46
Europe_1	3	48	4	55	1	23	4	9	14	4	55
Turkey	5	70	6	81				31	50		81
Africa		1		1	1						1
International.cultivar		11		11		9	1		1		11
(Europe = Europe_1+Turkey)	8	118	10	136	1	23	4	40	64	4	136
Total	57	183	28	268	16	35	38	64	109	6	268
Region	Percentage of accessions in each region by cluster (%) in the column										
	Based on two clusters				Based on five clusters						
	Q1	Q2	Admixture	sum	q1	q2	q3	q4	q5	Admixture	sum
Asia	66.7	12.0	50.0	27.6	87.5	2.9	13.2	23.4	33.9	33.3	27.6
America	19.3	16.9	14.3	17.2	0.0	5.7	73.7	14.1	6.4		17.2
Europe_1	5.3	26.2	14.3	20.5	6.3	65.7	10.5	14.1	12.8	66.7	20.5
Turkey	8.8	38.3	21.4	30.2				48.4	45.9		30.2
Africa		0.5		0.4	6.3						0.4
International.cultivar		6.0		4.1		25.7	2.6		0.9		4.1
(Europe = Europe_1+Turkey)	14.0	64.5	35.7	50.7	6.3	65.7	10.5	62.5	58.7	66.7	50.7
Total	100	100	100	100	100	100	100	100	100	100	100
Region	Percentage of accessions in each cluster by region (%) in the row										
	Based on two clusters				Based on five clusters						
	Q1	Q2	Admixture	sum	q1	q2	q3	q4	q5	Admixture	sum
Asia	51.4	29.7	18.9	100	18.9	1.4	6.8	20.3	50.0	2.7	100
America	23.9	67.4	8.7	100		4.3	60.9	19.6	15.2		100
Europe_1	5.5	87.3	7.3	100	1.8	41.8	7.3	16.4	25.5	7.3	100
Turkey	6.2	86.4	7.4	100				38.3	61.7		100
Africa		100		100						100	100
International.cultivar		100		100		81.8	9.1		9.1		100
(Europe = Europe_1+Turkey)	5.9	86.8	7.4	100	0.7	16.9	2.9	29.4	47.1	2.9	100
Total	21.3	68.3	10.4	100	6.0	13.1	14.2	23.9	40.7	2.2	100

**Fig 2:**
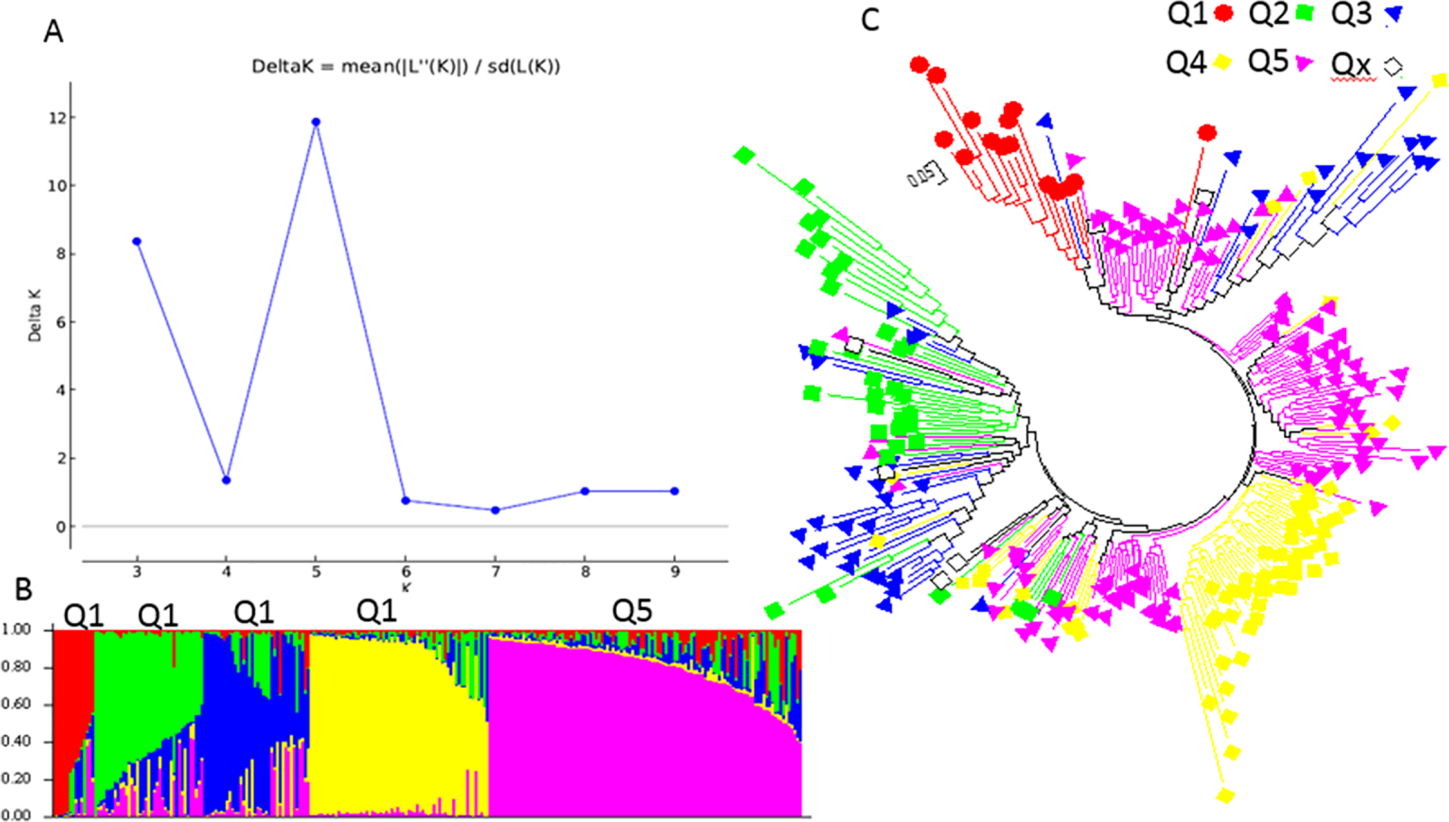
Model-based populations in association panels consisted of 268 USDA GRIN germplasm accessions: (a) Delta K values for different numbers of populations (K) assumed in analysis completed with the STRUCTURE software. (b) Classification of 268 spinach accessions into two populations using STRUCTURE Version 2.3.4, where the numbers on the y-axis show the subgroup membership, and the x-axis shows the different accession. The distribution of accessions into different populations is indicated by the color coding and shape (Cluster 1, Q1, is red round shape; Cluster 2, Q2, is green squared; Cluster 3, Q3, is blue triangle; Cluster 4, Q4, is yellow diamond; and Cluster 5, Q5, is purple triangle). (c) Maximum Likelihood (ML) tree of the 268 spinach accessions drawn in MEGA 6. The color code for each population is consistent in the (b) and (c), and the empty black square represents accessions aligned with the admixture cluster or population, in 268 USDA GRIN spinach germplasm accessions.

The genetic diversity among germplasm accessions was also assessed using the Maximum Likelihood (ML) method in MEGA 6 [34] (Tamura et al., 2013), with phylogenetic trees drawn based on the results. Q1 and Q2 were defined as the two main clusters or populations (see above), with the same colors as the population structure Q1 (red) and Q2 (green) from the STRUCTURE 2.3.4 analysis (Fig 1B) to draw subtrees of the phylogenetic tree (Fig 1C). Q1 is denoted with a red color and round shape, and Q2 with a green color and square shape. Two phylogenetic trees were included: (1) without taxon names assigned in order to compare the populations from STRUCTURE (Fig 1C), and (2) the traditional rectangular phylogenetic tree (S1 Fig). The phylogenetic trees from MEGA 6 (Fig 1C and S1 Fig), were well consistent with the structure populations Q1 and Q2 developing in STRUCTURE 2.3.4 (Fig 1A and 1B), indicating there were two differentiated genetic populations in the spinach germplasm panel, which was divided distinctly into two clusters.

Besides the two structured populations inferred using the STRUCTURE analysis, the second highest peak of delta K was observed at K = 5 using Structure Harvester, indicating the 268 spinach germplasm accessions may be divided into five clusters (Q1 to Q5) (Fig 2A). Fig 2B shows the bar plot drawn in STRUCTURE to visualize the five clustered populations, where Q1 is red; Q2 is green; Q3 is blue; Q4 is yellow; and Q5 is purple. The classification of the germplasm accessions into populations based on the model-based structure developed in STRUCTURE 2.3.4 was shown in Fig 2B and S2 Table. Each spinach accession also was assigned to one of the five populations based on probabilities calculated in STRUCTURE (S2 Table). A Q value = 0.5 was used to divide the five populations (clusters) and the admixture. In total, 262 out of 268 accessions (97.8%) were assigned to one of the five populations, Q1 to Q5. Q1 to Q5 were consisted of 16 (6.0%), 35 (13.1%), 38 (14.2%), 64 (23.9%), and 109 (40.7%) accessions, respectively. The remaining 6 accessions (2.2%) were categorized as having admixed ancestry between Q1 to Q5 (S2 Table). Based on the five structured populations or clusters Q1 to Q5 among the 268 spinach germplasm accessions, Q1 mainly consisted of Asia accessions with 14 out of 16 accessions having 87.5% of total Q1 accessions; Q2 mainly Europe but not Turkey accessions with 65.7%; Q3 America accessions with 73.7%; Q4 Turkey accessions with 48.4%; and Q5 also Turkey accessions with 45.5%. (Table 2), indicating there was a geographic effect existed for the spinach populations structured.

For the five populations (clusters), the genetic diversity of the 268 spinach accessions also was analyzed using the ML method in MEGA 6 by combining the five structured populations, Q1 to Q5, from STRUCTURE as done for the two structured populations above. The five clusters shown in Fig 2C were divided according to the five structured populations, Q1 to Q5, with same colors as in Fig 2B, indicating five differentiated genetic populations and admixtures among the 268 accessions. The same approaches and methods were also used to analyze genetic diversity of the five structured populations using MEGA 6. The two phylogenetic trees drawn were consistent with the structure populations from Q1 to Q5 from STRUCTURE 2.3.4, indicating there were five differentiated genetic subpopulations and admixtures in the accessions. However, the five structured populations were well clustered with exceptions (S2 Fig), where Q1 is red; Q2 is green; Q3 is blue; Q4 is yellow; and Q5 is purple; and the admixture of the five populations is represented by black empty squares.

### Genetic diversity in commercial F1 hybrids

**Fig 3:**
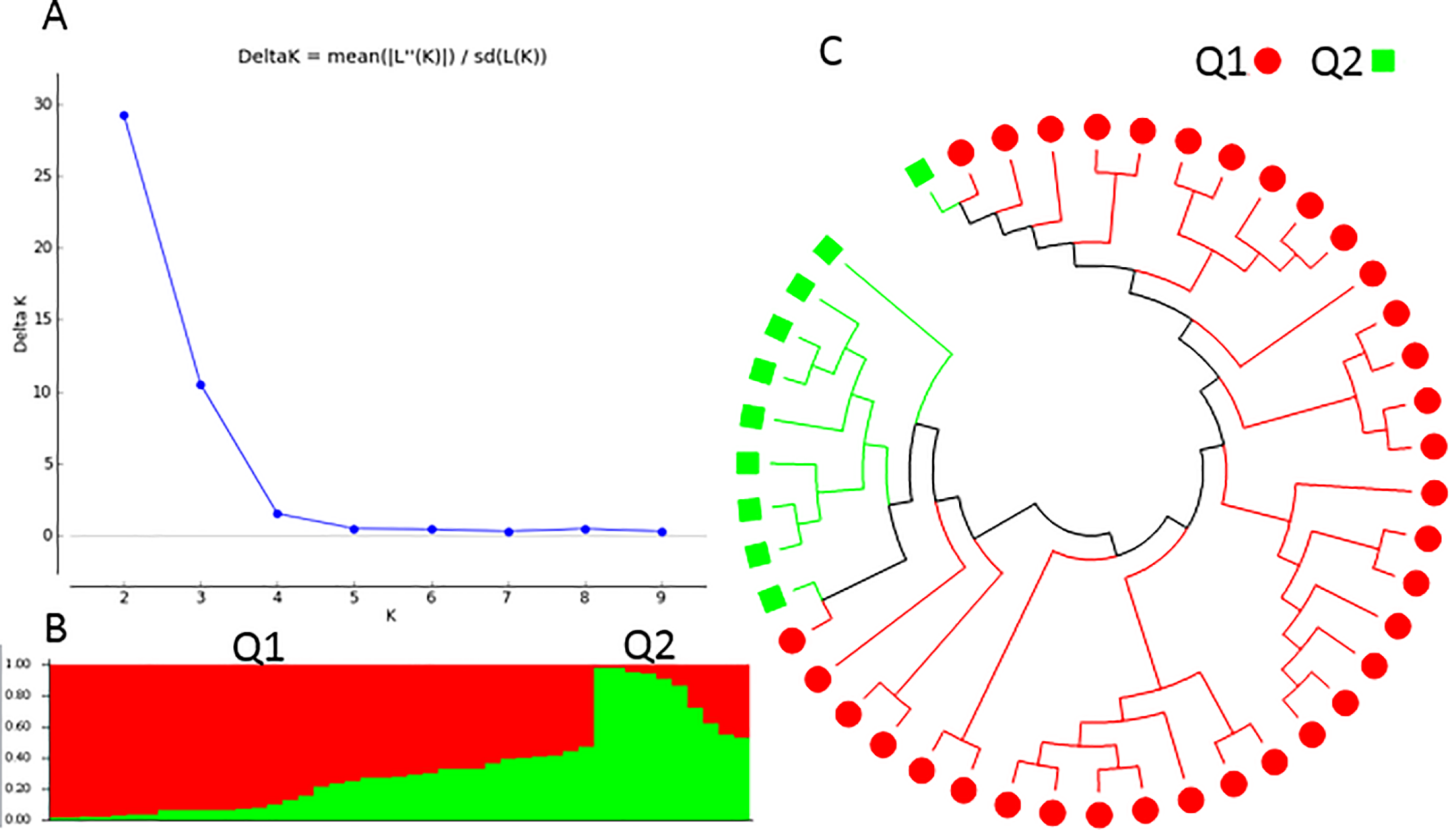
Model-based populations in association panels consisted of 45 F1 hybrids: (a) Delta K values for different numbers of populations (K) assumed in analysis completed with the STRUCTURE software. (b) Classification of 45 F1 hybrids into two populations using STRUCTURE Version 2.3.4, where the numbers on the y-axis show the subgroup membership, and the x-axis shows the different accession. The distribution of accessions into different populations is indicated by the color coding and shape (Cluster 1, Q1, is red round shape; and Cluster 2, Q2, is green squared). (c) Maximum Likelihood (ML) tree of the 45 F1 hybrids drawn in MEGA 6. The color code for each population is consistent in the (b) and (c), and the empty black square represents accessions aligned with the admixture cluster or population, Q1Q2.

The population structure of the 45 commercial F1 hybrids was initially inferred using STRUCTURE 2.3.4 (Pritchard et al., 2000) with the same approach as we did for the USDA germplasm set. The peak delta K was observed at K = 2, indicating the presence of two main population clusters, Q1 and Q2, in the F1 spinach panel (Fig 3A and 3B). The classification of accessions into populations or clusters from the model-based structure using STRUCTURE 2.3.4 is shown in Fig 3B and S3 Table. In total, all 45 F1 hybrids (100%) were assigned to one of the two populations or clusters. Q1 and Q2 consisted of 35 (77.8%) and 10 (22.2%) F1 hybrids, respectively (S3 Table).

The genetic diversity among spinach F1 hybrids was also assessed using the Maximum Likelihood (ML) method in MEGA 6 (Tamura et al., 2013), with phylogenetic trees drawn based on the results. Q1 and Q2 were defined as the two main clusters or populations (see above), with the same colors as the population structure Q1 (red) and Q2 (green) from the STRUCTURE analysis (Fig 3B) to draw subtrees of the phylogenetic tree (Fig 3C). Q1 is denoted with a red color and round shape, and Q2 with a green color and square shape. Two phylogenetic trees were included: (1) without taxon names assigned in order to compare the populations from STRUCTURE (Fig 3C), and (2) the traditional rectangular phylogenetic tree (S3 Fig). The phylogenetic trees from MEGA 6 (Fig 3C and S3 Fig), were well consistent with the structure populations Q1 and Q2 from in STRUCTURE 2.3.4 (Fig 3A and 3B), indicating that there were two differentiated genetic populations in the spinach F1 panel, which was divided distinctly into two clusters with exceptions. The spinach hybrid ‘Whale F1’ was grouped into Cluster Q2 based on STRUCTURE, but it was located at cluster I based on MEGA 6.

**Fig 4:**
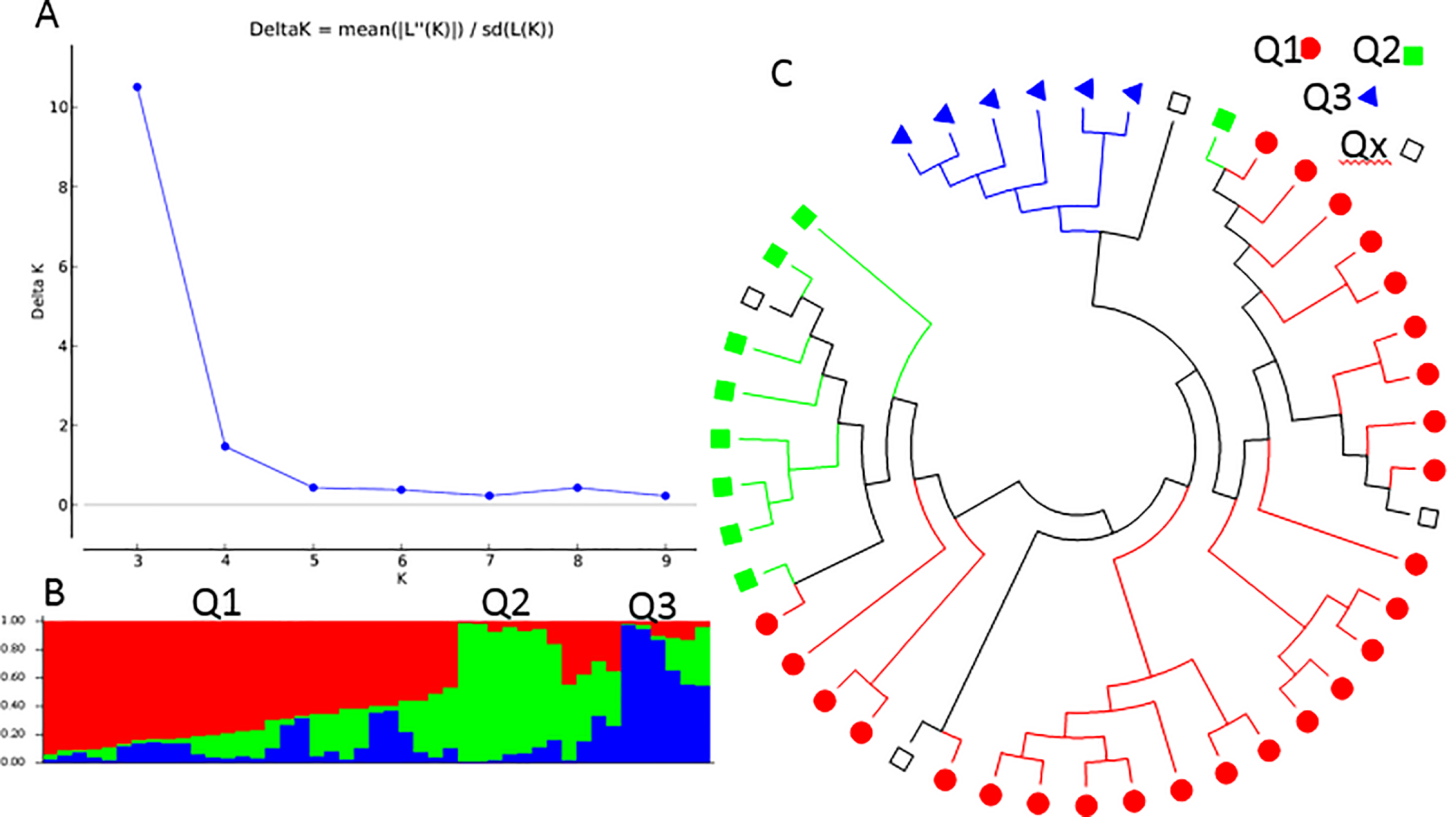
Model-based populations in association panels consisted of 45 F1 hybrids: (a) Delta K values for different numbers of populations (K) assumed in analysis completed with the STRUCTURE software. (b) Classification of 45 F1 hybrids into two populations using STRUCTURE Version 2.3.4, where the numbers on the y-axis show the subgroup membership, and the x-axis shows the different accession. The distribution of accessions into different populations is indicated by the color coding and shape (Cluster 1, Q1, is red round shape; Cluster 2, Q2, is green squared; and Cluster 3, Q3, is blue triangle). (c) Maximum Likelihood (ML) tree of the 45 F1 hybrids drawn in MEGA 6. The color code for each population is consistent in the (b) and (c), and the empty black square represents accessions aligned with the admixture cluster or population.

Besides the two structured populations inferred using the STRUCTURE analysis, the second highest peak of delta K was observed at K = 3 using Structure Harvester, indicating the 45 spinach F1 hybrids can be divided into three population clusters (Q1 to Q3) (Fig 4A). Fig 4B shows the bar plot drawn in STRUCTURE to visualize the three populations, where Q1 is red; Q2 is green; Q3 is blue; and the admixture of the three populations is represented by black empty squares. The classification of F1 hybrids into populations from the model-based structure developed in STRUCTURE 2.3.4 is shown in Fig 4B and S3 Table. Each spinach F1 hybrid was also assigned to one of the three populations based on probabilities calculated in STRUCTURE (S3 Table). A Q value = 0.5 was used to divide the three clusters and the admixture. In total, 41 out of 45 F1 hybrids (91.1%) were assigned to one of the three populations, Q1 to Q3. Q1 to Q3 consisted of 26 (57.8%), 9 (20.0%), and 6 (13.3%) F1 hybrids, respectively. The remaining 4 hybrids (8.9%) were categorized as having admixed ancestry between Q1 to Q3 (S3 Table).

The genetic diversity of the 45 spinach accessions also was analyzed using the ML method in MEGA 6 by combining the three structured populations, Q1 to Q3, from STRUCTURE as done for the two structured populations above. The three clusters shown in Fig 4C were divided according to the three structured populations, Q1 to Q3, with same colors as in Fig 4B, indicating three differentiated genetic populations and admixtures among the 45 F1 hybrids. The same approaches and methods were also used to analyze genetic diversity of the three structured populations using MEGA 6. The two phylogenetic trees drawn were consistent with the structure populations Q1 to Q3 from STRUCTURE, indicating that there were three differentiated genetic subpopulations and admixtures in the F1 hybrids panel. However, the three structured subpopulations were well clustered with exception (S4 Fig). The Whale F1 still was classified into Cluster Q2 based on STRUCTURE 2, but it was located at cluster I based on MEGA 6.

Among the 45 F1 hybrids, three F1 hybrids were developed by the company from Asia, 33 from Europe, and nine from America. Based on genetic diversity analysis by MEGA 6, the diversity was not related to geography of content. Based on the seeds developed by companies, the F1 hybrids from the same company were merged together, indicating that the F1 hybrids from the same company have the similarity in genetic backgrounds (S3 and S4 Figs).

### Genetic diversity by region (group)

Based on geography in spinach panel, seven regions were generated in this study: ‘America’, ‘Asia’, ‘Europe’, ‘Turkey’, ‘Netherlands Commercial’, ‘America Commercial’ and ‘US Arkansas’. ‘America’ is the group of spinach accessions which were originally collected from United States of America (USA); ‘Asia’ is the group of the spinach accessions which were originally collected from Asia; ‘Europe’ is the group of the spinach accessions which were originally collected from Europe except Turkey; ‘Turkey’ is the group of the spinach accessions which were originally collected from Turkey (*because there were 81 spinach accessions from Turkey consisted of 30.2% of all 268 accessions, we listed Turkey as one group out of the Europe group.); ‘Netherlands Commercial’ is the group of the spinach commercial F1 hybrids which were originally collected from Netherlands; ‘‘America Commercial’ is the group of the spinach commercial F1 hybrids which were originally collected from Unites States of America; and ‘US Arkansas” is the group of the spinach lines which were originally collected from US Arkansas. In this study, we have only two sources of commercial F1 hybrids: one from Netherlands and another from USA.

**Fig 5:**
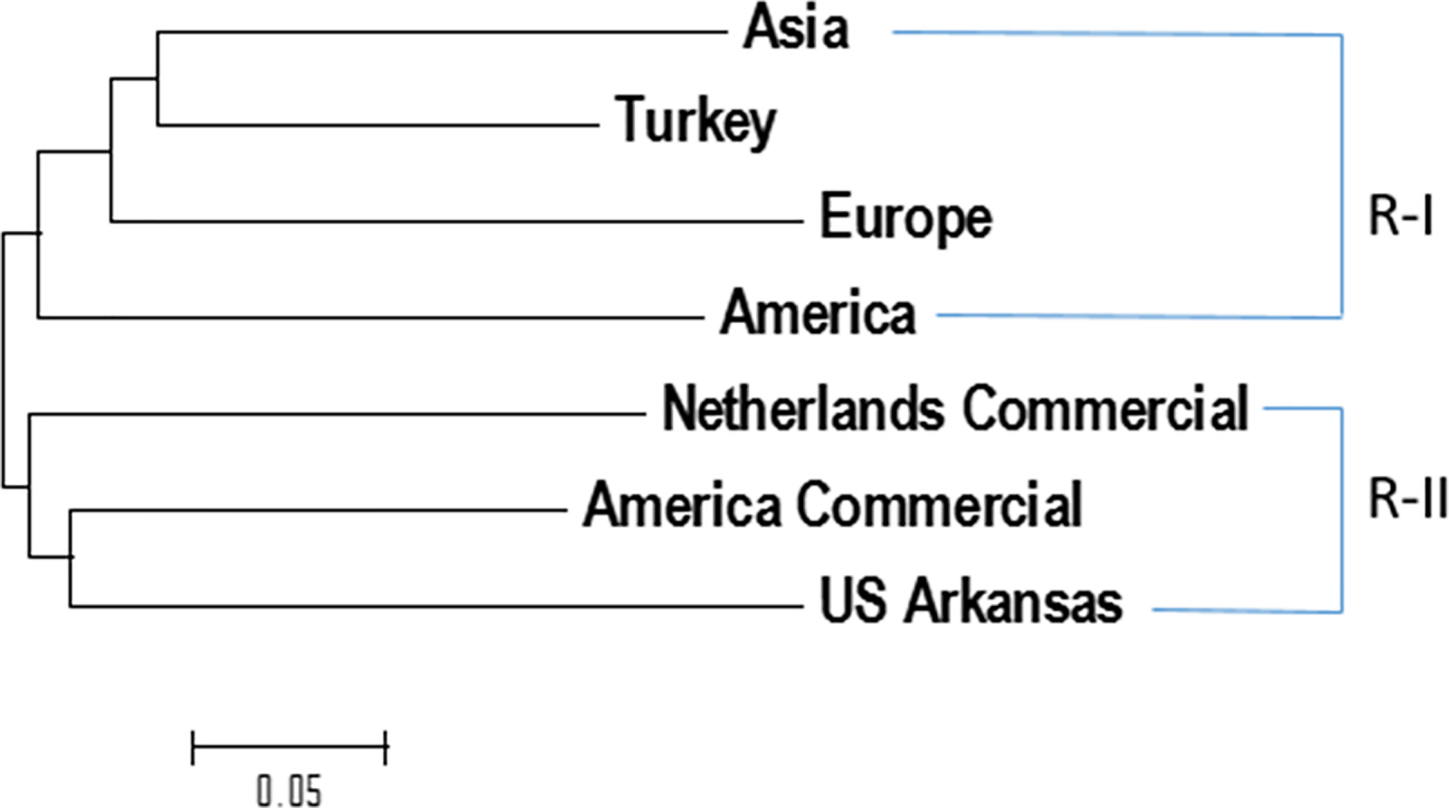
The traditional phylogenetic tree created by the Maximum Likelihood (ML) method from MEGA 6 in spinach genotypes from 7 regions (groups).

The genetic diversity among the seven regions were analyzed and phylogenetic tree was drawn using MEGA 6: first computed between group mean distances and a two dimensions of genetic distances among the seven regions was created; and then the phylogenetic tree was drawn using neighbor-joining method. The results showed that (1) two clusters were observed (Fig 5): Cluster R-I consisted of all spinach germplasm accessions from Asia, Turkey, Europe, and America. Cluster R-II included all commercial hybrids (Netherlands Commercial and America Commercial) and the Arkansas lines; (2) the spinach accessions from Turkey are closest to those from Asia, then closer to those from Europe, and then to America; and (3) both commercial F1 hybrids were closer to each other, indicating that the commercial F1 hybrids had closer genetic background than those germplasm. But they were merged together with US Arkansas spinach lines, validating that the Arkansas spinach lines have provided genetic resource used in both American and Netherland F1 hybrids as parents.

### Genetic diversity by country

**Fig 6:**
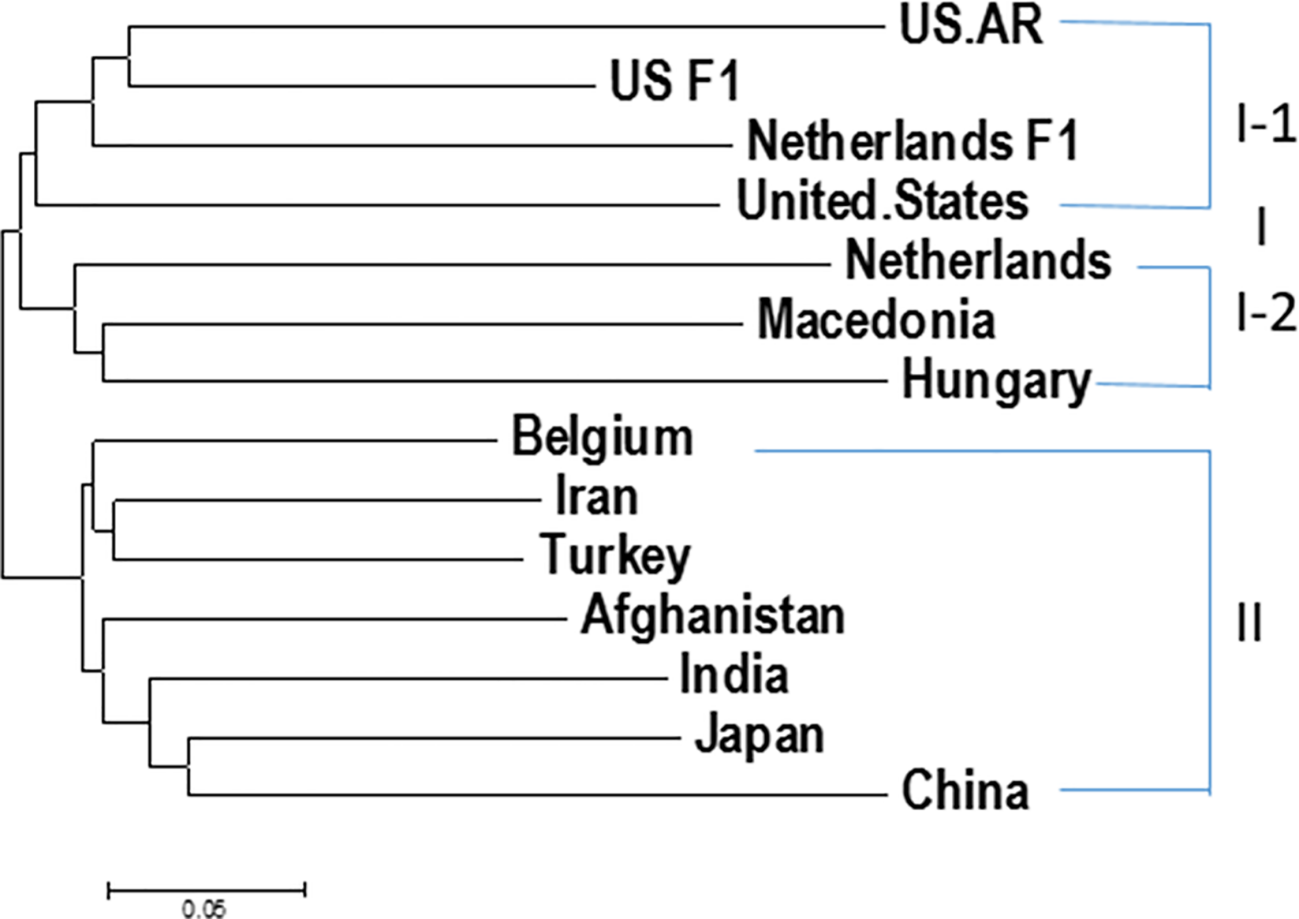
The traditional phylogenetic tree created by the Maximum Likelihood (ML) method from MEGA 6 in spinach germplasm accessions from 12 countries plus F1 hybrids and Arkansas lines.

There are 11 countries with 6 or more spinach accessions in this study: Afghanistan has 15 accessions, Belgium 9, China 20, Hungary 6, India 6, Iran 15, Japan 6, Macedonia 16, Netherlands 36 (4 germplasm accessions and 32 hybrids), Turkey 81, and United States 81 (46 US germplasm accessions, 5 F1 and 30 breeding lines developed by University of Arkansas) (Tables 1, 2, and 3, and S1 and S4 Tables). The genetic diversity was analyzed for those spinach genotypes from the 11 countries in this study. In order to distinguish the sources of germplasm, commercial hybrids and Arkansas lines, the spinach genotypes from Netherlands were divided into two groups: Netherlands germplasm (‘Netherlands’) and Netherlands F1, and the spinach from America divided into three groups: America germplasm (‘United.States’), America F1 hybrids (‘US F1’), and Arkansas lines (‘US.AR’). In that way, a total of 14 ‘country groups’ were formed for genetic diversity study. Phylogenetic analysis showed that two larger clusters were produced: Cluster I included the spinach accessions from Arkansas lines (US.AR), commercial F1 hybrids from United States (US F1) and from Netherland (Netherlands F1), Unites States, Netherlands, Macedonia, and Hungary, and Cluster II consisted of the spinach germplasm accessions from Belgium Iran, Turkey, Afghanistan, India, Japan, and China. The cluster I can be further divided into two cluster I-1 and I-2 (Fig 6). Cluster I-1 included the accessions from US.AR, US F1, Netherland F1, and Unites States. Cluster I-2 had the accessions from Netherlands, Macedonia, and Hungary. The results furtherly indicated that spinach germplasm accessions had geographic effect that all accessions from Asia (Iran, Afghanistan, India, China, and Japan) were merged into cluster II and the accessions from the three European countries (Netherland, Macedonia, and Hungary) merged into cluster I-2. Both commercial F1 hybrids from United States (US F1) and from Netherlands (Netherlands F1) merged together, indicating that the commercial hybrids had closer genetic background than those germplasm. Both commercial F1 hybrids either from United States (US F1) or from Netherlands (Netherlands F1) merged together with Arkansas spinach lines (US.AR), indicating that the spinach germplasm from the University of Arkansas has been used in spinach commercial as parents and the commercial F1 hybrids have inheritable some genetic backgrounds from the Arkansas germplasm.

**Table 3:** Three parameters were estimated: (1) The number of spinach accessions in each cluster was list by region (up section in the table); (2) the percentage of accessions in each cluster was listed by region and the data were shown in each row (the middle section in the table); and (3) the percentage of accessions in each region was listed by cluster and the data were shown in each column (bottom section in the table) in the 343 world wide spinach genotypes including 268 USDA accessions, 45 F1 hybrids and 30 Arkansas lines.

**Group**	**Region**	**No. of accessions in each cluster by region**									
		**Based on two clusters**				**Based on four clusters**					
		**Q1**	**Q2**	Admixture	**sum**	**Q1**	**Q2**	**Q3**	**Q4**	Admixture	**sum**
**F1 hybrids**	Asia.America	2		1	3			1	1	1	3
	Europe	13	16	4	33	4	1		27	1	33
	America?	1	2	1	4		1		3		4
	America	3	2		5	1	1		2	1	5
	F1 subtotal	19	20	6	45	5	3	1	33	3	45
**Arkansas** **spinach**	AR		30		30		30				30
**USDA-GRIN spinach germplasm**	Asia	74			74	50		18	2	4	74
	America	38	6	2	46	23			21	2	46
	Europe_1	54	1		55	47		1	6	1	55
	Turkey	81			81	80				1	81
	Africa	1			1					1	1
	International.cultivar	11			11	10			1		11
	Germplasm subtotal	259	7	2	268	210		19	30	9	268
	(Europe = Europe_1+Turkey)	135	1		136	127		1	6	2	136
	Total	278	57	8	343	215	33	20	63	12	343
**Group**	Region	Percentage of accessions in each region by cluster (%) in the column									
		Based on two clusters				Based on five clusters					
		Q1	Q2	Admixture	sum	Q1	Q2	Q3	Q4	Admixture	sum
**F1 hybrids**	Asia.America	0.7	0.0	12.5	0.9			5.0	1.6	8.3	0.9
	Europe	4.7	28.1	50.0	9.6	1.9	3.0		42.9	8.3	9.6
	America?	0.4	3.5	12.5	1.2		3.0		4.8		1.2
	America	1.1	3.5		1.5	0.5	3.0		3.2	8.3	1.5
	F1 sum	6.8	35.1	75.0	13.1	2.3	9.1	5.0	52.4	25.0	13.1
**Arkansas** **spinach**	AR		52.6		8.7		90.9				8.7
**USDA-GRIN spinach germplasm**	Asia	26.6			21.6	23.3		90.0	3.2	33.3	21.6
	America	13.7	10.5	25.0	13.4	10.7			33.3	16.7	13.4
	Europe	19.4	1.8		16.0	21.9		5.0	9.5	8.3	16.0
	Turkey	29.1			23.6	37.2				8.3	23.6
	Africa	0.4			0.3					8.3	0.3
	International.cultivar	4.0			3.2	4.7			1.6		3.2
	Germplasm subtotal	93.2	12.3	25.0	78.1	97.7		95.0	47.6	75.0	78.1
	(Europe = Europe_1+Turkey)	48.6	1.8		39.7	59.1		5.0	9.5	16.7	39.7
	Total	100	100	100	100	100	100	100	100	100	100
**Group**	Region	Percentage of accessions in each cluster by region (%) in the row									
		Based on two clusters				Based on four clusters					
		Q1	Q2	Admixture	sum	Q1	Q2	Q3	Q4	Admixture	sum
**F1 hybrids**	Asia.America	66.7	0.0	33.3	100	0.0	0.0	33.3	33.3	33.3	100
	Europe	39.4	48.5	12.1	100	12.1	3.0		81.8	3.0	100
	America?	25.0	50.0	25.0	100		25.0		75.0		100
	America	60.0	40.0		100	20.0	20.0		40.0	20.0	100
	sum	42.2	44.4	13.3	100	11.1	6.7	2.2	73.3	6.7	100
**Arkansas spinach**	AR		100		100		100				100
**USDA-Grin** **Spinach germplasm**	Asia	100			100	67.6		24.3	2.7	5.4	100
	America	82.6	13.0	4.3	100	50.0			45.7	4.3	100
	Europe_1	98.2	1.8		100	85.5		1.8	10.9	1.8	100
	Turkey	100			100	98.8				1.2	100
	Africa	100			100					100.0	100
	International.cultivar	100			100	90.9			9.1		100
	Germplasm subtotal	96.6	2.6	0.7	100	78.4		7.1	11.2	3.4	100
	(Europe = Europe_1+Turkey)	99.3	0.7		100	93.4		0.7	4.4	1.5	100
	Total	81.0	16.6	2.3	100	62.7	9.6	5.8	18.4	3.5	100

### Genetic diversity in United States

A total of 91 spinach genotypes collected from United States were used for genetic diversity analysis in this study. Eighty-one of the 91 spinach genotypes had clear originally from US, which includes 46 US germplasm accessions, 5 US hybrids, and 30 breeding lines developed by University of Arkansas (Tables 1, 2 and 3, and S1 and S4 Tables). In additions, three F1 hybrids, Alrite F1, Megaton F1, and Summer Focus F1 were from American Takii Company and the original seeds may be developed from America but they may be also developed in Japan. Four hybrids, Denali F1, Hector.F1, Indian Summer F1, and Spinner F1 were from Johnny Selected Seeds and the original seeds may be developed from America. The seven hybrids from American Takii and Johnny Selected Seeds were included in phylogenetic analysis. Besides the 04-103VGRS from AR lines, three its sister lines, 04-103FAY, 04-103FAY_09, and 04-103VGRS_09 were also included in the phylogenetic analysis with a total of 91 spinach genotypes (S5 Fig). From the phylogenetic tree, all 33 AR lines including the three 04-103VGRS sister-line were merged together as one group. Among which the 04-103VGRS and its three its sister lines were close together (S5 Fig); the 12 F1 hybrids were distributed in the two locations of the phylogenetic tree, among which Denali F1, Regal F1, Samish F1, Coho F1, and both Nordic IV F1 were closed together and near AR lines; and Indian Summer F1, Spinner F1, Tyee F1, Olympia F1, and Hector F1 closed together in another group; and most of PIs from US Maryland merged together. These results indicating that (1) Arkansas spinach had specific genetic backgrounds which separated from others; (2) hybrids were different from the germplasm as they merged together with two locations; and (3) there was a partially geographic effect because many spinach accessions were clustered by their origin of States.

### Genetic diversity in Arkansas lines

The major contributions of the spinach breeding program at the University of Arkansas have been in the areas of disease resistant cultivars, particularly to white rust, such as ‘Fallgreen’, released in 1987, ‘F380’ released in 1998, and ‘Wintergreen’ released in 2003. These are some of the most highly resistant genotypes to white rust, and have been used widely as germplasm for white rust resistance and as parents by seed companies to develop resistant hybrids. Though the AR spinach germplasm had a relatively narrow genetic background compared to the world-wide spinach germplasm variation, the 30 AR spinach lines can be divided into four groups: AR-I, consisted of 18 lines including 08–116, 08–122, and 97–154; AR-II, comprised four lines, 04-103VGRS, 08-88-212, 08–104, and 08–140; AR-III, having only two lines, 08-03-318 and 08–101; and AR-IV, containing 6 lines including 08–144, 08–150, 08F380, 08–167, and 08–191 (S6 Fig), indicating that some variation among the Arkansas lines.

### Genetic diversity in world-wide spinach genotypes

As described above, a total of 343 spinach genotypes were used in this study, which included three types of spinach resources: 268 USDA GRIN spinach germplasm accessions, 45 spinach F1 hybrids, and 30 US Arkansas spinach lines (Table 1). The genetic diversity and population structure have been analyzed for the 268 USDA accessions, the 45 hybrids, and the 30 Arkansas lines, respectively. Based on the three resources, a combined analysis of genetic diversity and population structure were conducted for the 343 spinach genotypes in this study as well.

**Fig 7:**
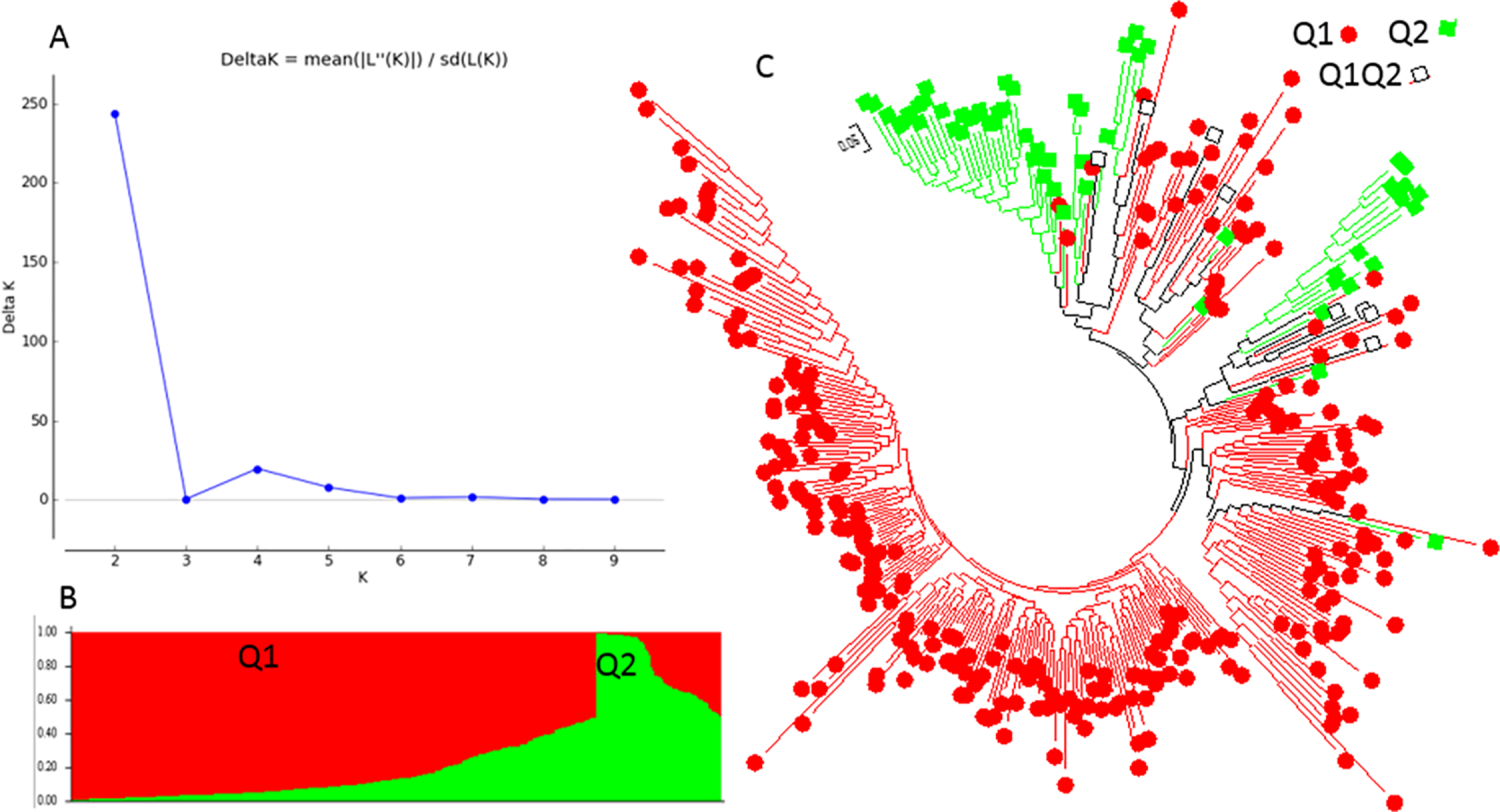
Model-based populations in association panels consisted of 343 spinach genotypes: (a) Delta K values for different numbers of populations (K) assumed in analysis completed with the STRUCTURE software. (b) Classification of 343 spinach genotypes into two populations using STRUCTURE Version 2.3.4, where the numbers on the y-axis show the subgroup membership, and the x-axis shows the different accession. The distribution of accessions into different populations is indicated by the color coding and shape (Cluster 1, Q1, is red round shape; and Cluster 2, Q2, is green squared). (c) Maximum Likelihood (ML) tree of the 343 spinach genotypes drawn in MEGA 6. The color code for each population is consistent in the (b) and (c), and the empty black square represents accessions aligned with the admixture cluster or population, Q1Q2.

As we did for the 268 USDA accessions, the population structure of the 343 spinach genotypes was initially inferred using STRUCTURE 2.3.4 (Pritchard et al., 2000). The peak delta K was observed at K = 2, indicating the presence of two main population clusters, Q1, and Q2 in the spinach panel (Fig 7A and 7B). The classification of accessions into populations or clusters according to the model-based structure from STRUCTURE 2.3.4 is shown in Fig 7B and S1 Table. A Q-value = 0.50 was used to divide the clusters. In total, 335 of 343 germplasm accessions (97.7%) were assigned to one of the two populations or clusters. Q1 and Q2 consisted of 278 (81.0%) and 57 (16.6%) spinach genotypes, respectively, and the remaining 8 accessions (2.3%) were categorized as having admixed ancestry between Q1Q2 (Table 3, S1 Table). In the Q2 structured population, 50 out of 57 (87.7%) spinach genotypes were the Arkansas lines (30) and F1 commercial hybrids (20) (Table 3), and the two populations distinguished the USDA germplasm accessions from the commercial F1 hybrids and Arkansas lines, indicating that the commercial F1 hybrids and Arkansas lines have improved with their genetic backgrounds. Analyses based on each region-typed showed that the germplasm accessions except America mainly went to Q1; America had 13% in Q2 and 4.4% in the mixture Q1Q2; Arkansas went to Q2 with 100%; and hybrids went to both Q1, Q2, and mixture with 42.2%, 44.4%, and 13.3%, respectively, furtherly indicating that Arkansas lines had different genetic background from germplasm accessions; hybrids had both genetic backgrounds; and the majority of American germplasm accessions had more similar genetic background to other germplasm but were closer to the hybrid than others.

The genetic diversity among 343 genotypes was also assessed using the Maximum Likelihood (ML) method in MEGA 6 (Tamura et al., 2013), with phylogenetic trees drawn based on the results. Q1 and Q2 were defined as the two main clusters or populations (see above), with the same colors as the population structure Q1 (red) and Q2 (green) from the STRUCTURE 2.3.4 analysis (Fig 7B) to draw subtrees of the phylogenetic tree (Fig 7C). Q1 is denoted with a red color and round shape, and Q2 with a green color and square shape. Two phylogenetic trees were included: without taxon names assigned in order to compare the populations from STRUCTURE (Fig 7C), and the traditional rectangular phylogenetic tree (S7 Fig). The phylogenetic trees from MEGA 6 (Fig 7C and S7 Fig), were well consistent with the structure populations Q1 and Q2 developed in STRUCTURE 2.3.4 (Fig 7A and 7B), indicating that there were two differentiated genetic populations in the 343 spinach genotypes panel, which was divided distinctly into two clusters with exceptions.

**Fig 8:**
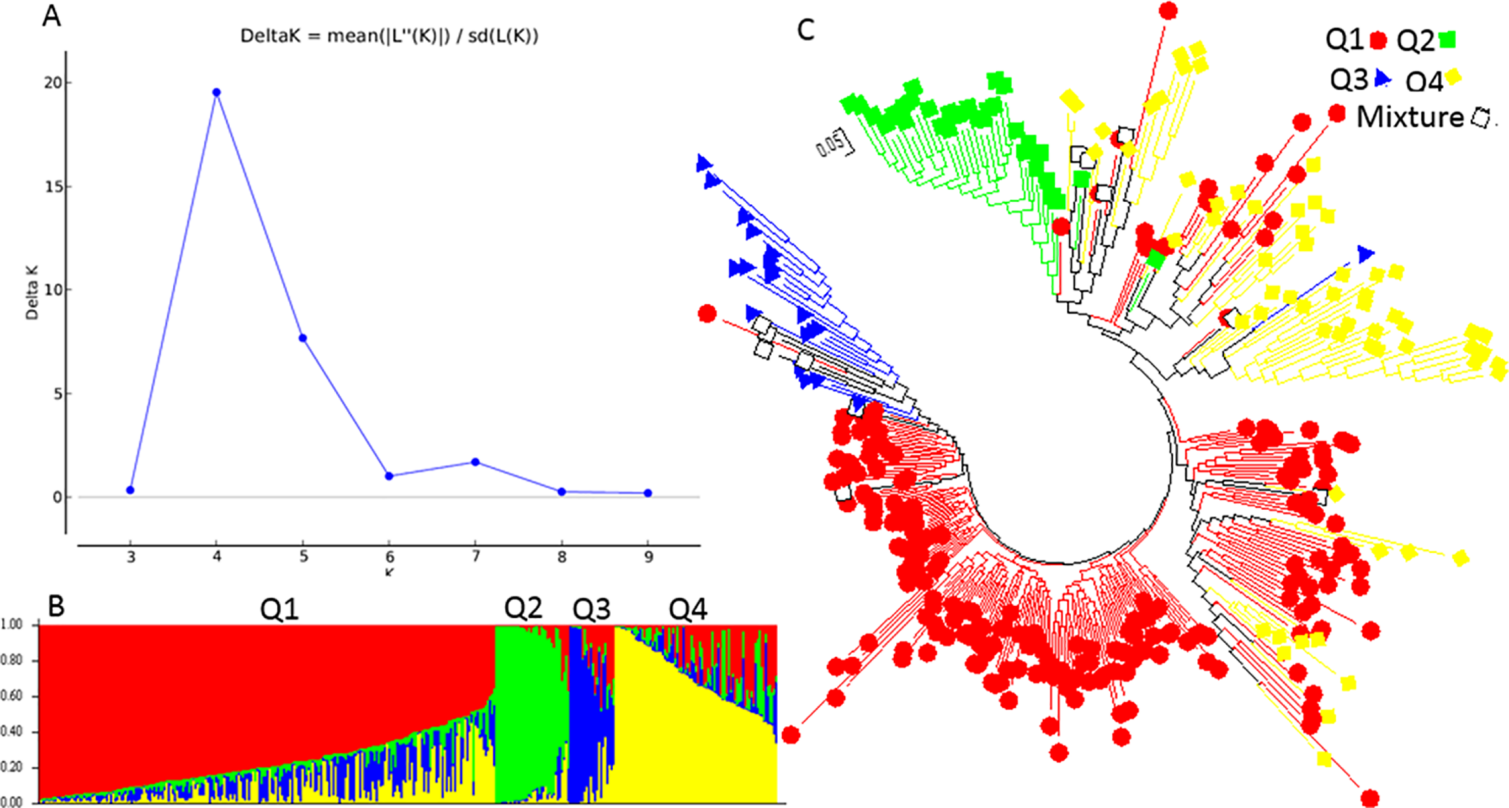
Model-based populations in association panels consisted of 343 spinach genotypes: (a) Delta K values for different numbers of populations (K) assumed in analysis completed with the STRUCTURE software. (b) Classification of 343 spinach genotypes into two populations using STRUCTURE Version 2.3.4, where the numbers on the y-axis show the subgroup membership, and the x-axis shows the different accession. The distribution of accessions into different populations is indicated by the color coding and shape (Cluster 1, Q1, is red round shape; Cluster 2, Q2, is green squared; Cluster 3, Q3, is blue triangle; and Cluster 4, Q4, is yellow diamond). (c) Maximum Likelihood (ML) tree of the 343 spinach genotypes drawn in MEGA 6. The color code for each population is consistent in the (b) and (c), and the empty black square represents accessions aligned with the admixture cluster or population, in 343 spinach genotypes.

Besides the two structured populations inferred using the STRUCTURE analysis, the second highest peak of delta K was observed at K = 4 using Structure Harvester, indicating the 343 spinach genotypes can be also divided into four population clusters (Q1 to Q4) (Fig 8A). Fig 8B shows the bar plot drawn in STRUCTURE to visualize the three populations, where Q1 is red; Q2 is green; Q3 is blue; Q4 is yellow, and the admixture of the three populations is represented by black empty squares. The classification of the germplasm accessions into populations according with the model-based structure developed in STRUCTURE 2.3.4 was shown in Fig 8B and S1 Table. Each spinach genotype was also assigned to one of the four populations based on probabilities calculated in STRUCTURE (S1 Table). A Q value = 0.5 was used to divide the three clusters and the admixture. In total, 331 out of 343 accessions (96.5%) were assigned to one of the four populations, Q1 to Q4. Q1 to Q4 consisted of 215, 33, 20, and 63 accessions with 62.7%, 9.6%, 5.8%, and 18.4%, respectively. The remaining 12 accessions (3.5%) were categorized as having admixed ancestry between Q1 to Q4 (Table 3, S1 Table). Based on the four structured populations (Q1 to Q4), Q1 consisted of mainly germplasm accessions (97.7%); Q2 included the Arkansas lines (90.9%) and hybrids F1 (9.1%) without any germplasm accession; Q3 was mainly from Asia (90%); and Q4 had mainly F1 (52.4%) and America germplasm (33.3%) (Table 3), indicating that Arkansas lines and hybrids had different genetic background and formed one cluster Q2; Asia accessions had special genetic background different from others; and the commercial hybrids had the genetic backgrounds closer Arkansas lines or America germplasm. For each grouped region, the majority of Asia accessions was Q1 with 67.6% but it had a quarter (24.3%) go to Q3; the America had half to Q1 and half to Q4; almost all Turkey (98.8%) and majority of Europe (93.4%) to Q1 (Table 3), indicating that the spinach population structure was associated with grouped regions and further showed that there was a geographic effect for spinach genetic diversity.

The genetic diversity of the 343 spinach genotypes was also analyzed using the ML method in MEGA 6 by combining the four structured populations, Q1 to Q4, from STRUCTURE as done for the four structured populations above. The four clusters shown in Fig 8C were divided according to the four structured populations, Q1 to Q4, with same colors as in Fig 8B, indicating four differentiated genetic populations and admixtures among the 343 spinach genotypes. The same approaches and methods were also used to analyze genetic diversity of the four structured populations using MEGA 6. The two phylogenetic trees drawn were consistent with the structure populations Q1 to Q4 from STRUCTURE 2.3.4, indicating that there were four differentiated genetic subpopulations and admixtures in the spinach set (S8 Fig).

## Discussion

In this study, three collections of spinach germplasm was used: 268 USDA GRIN spinach germplasm accessions originally collected from 30 countries, 45 commercial spinach F1 hybrids from three countries, and 30 US Arkansas spinach cultivars and breeding lines (Tables 1, 2 and 3, and Supplementary S1, S2, S3 and S4 Tables). The results from this study showed that there was genetic variations among the 343 spinach genotypes examined. The data indicated that the genetic diversity and population structures were associated with geography–region and country; and the US Arkansas spinach cultivars/lines had a unique genetic background.

### Genetic variation and population structure

In this study, spinach population structure was examined among 343 world-wide spinach genotypes from three spinach collections using the Markov Chain Monte Carlo iterations in STRUCTURE 2. The structured populations (clusters) were determined by the maximum value of the delta K and also the second greatest value (the second highest peak) of delta K was also used to access the populations. At the same time, the structured populations (clusters) from STRUCTURE 2 were confirmed by the ML method in MEGA 6.

The 268 USDA GRIN spinach germplasm accessions can be divided into two larger structured populations (Q1 and Q2 clusters plus the admixture) or into five structured subpopulations (Q1 to Q5 plus admixture). Based on the two broader structured populations (Q1 and Q2) in the 268 USDA germplasm accessions, Q1 consisted of germplasm mainly from Asia and the Q2 from Europe. The majority of America germplasm belonged to Q2.

Based on the demarcation of the five structured populations (Q to Q5), Asia material largely belonged to Q1; America to Q3; European (excluding Turkey) to Q2; Turkey to Q4 and other material from Europe to Q5, respectively (Table 2 middle). Regardless of the resolution for the 268 spinach germplasm accessions as two or as five structured populations or clusters, the majority of Asia accessions went to a distinct cluster separately from the accessions from America and Europe. In addition, the five structured populations (clusters) can distinguish America from others because 60.9% of Q3 were America and also 73.7% of America was grouped to Q3 (Table 3 bottom). We also observed that the majority of Q2 was Europe without any from Turkey, indicating that the Turkey spinach genotypes had different genetic background from other Europe genotypes (Table 3 bottom).

The 45 F1 commercial hybrids could be grouped into two or three structured populations (clusters) (Figs 3 and 4, S3, and S4 Figs, Table 3 and S3 Table) and the results showed these F1 hybrids from the various companies had similarity genetic backgrounds (S3 and S4 Figs), but each cluster had spinach genotypes from different companies.

There were two broader or four structured populations (clusters) among the 343 world-wide spinach genotypes tested (Figs 7 and 8, and Supplementary S7 and S8 Figs, and Table 3 and S1 Table). Regardless if there were two or four clusters demarcated, the commercial F1 hybrids and the Arkansas lines were grouped into different clusters from the USDA germplasm. Therefore, the genetic backgrounds in commercial F1 hybrids and in Arkansas cultivar/lines have been improved for cultivation and the F1 hybrids and Arkansas lines were merged together and separately from the USDA germplasm, which was also confirmed by the phylogenetic analysis from regions and countries (Figs 5 and 6). The population structure analysis also demarcated the hybrids and Arkansas lines from USDA GRIN germplasm (Table 3) where both F1 and Arkansas lines made up 87.7% (50/57) of Q2 if two clusters were demarcated and also made up 100% in Q2 if four clusters (populations) were demarcated.

For USDA spinach germplasm, we did population structure analysis and had phylogenetic trees among around 300 accessions during our association studies for leafminer (*Liriomyza langei*) resistance [36], oxalate concentration in leaf [37], leaf traits [38], and Stemphylium leaf spot (*Stemphylium botryosum* f. sp. *spinacia*) [39] using SNPs from GBS approach and aimed to create the Q-matrix to be used in TASSEL for association analysis. Shi and Mou (2016) [36] accessed 300 USDA spinach germplasm for their population structure using 783 SNPs from GBS and two structured populations were postulated and used for association analysis of leafminer (*Liriomyza langei*) resistance. Shi et al. (2016) [37] also had two structured populations among 300 USDA spinach germplasm estimated using 841 SNPs for association study in oxalate concentration. Ma et al. (2016) [38] postulated five structured populations among 323 USDA spinach germplasm accessions using 4077 SNPs and the five structured populations were used for association analysis of leaf traits, surface texture (smooth, savoy or semisavoy), petiole color (different shades of green vs. purple) and edge shape (serrate vs. entire) in spinach. Shi et al. (2016) [39] accessed 273 USDA spinach germplasm accessions using 787 SNPs and both two and five structured populations were estimated and used for association analysis for Stemphylium leaf spot (*Stemphylium botryosum* f. sp. *spinacia*) resistance in spinach. In previous studies, both two and five structured populations in USDA germplasm accessions were postulated and used in association studied [36–39], similar to the results in this research. In additions, the population structure and phylogeny analysis were also conducted by regions and country in current research.

Currently, there are around 400 spinach germplasm accessions available in USDA GRIN and we plan to do whole genome resequencing (WGR) with 6X coverage of the spinach genome in the 400 accessions. Besides the AYZV02, the new released spinach genome LZYP01 (https://www.ncbi.nlm.nih.gov/Traces/wgs/LZYP01) with 88% genome coverage, and newest one in Allen van Deynze’s lab in UC Davis with six assembly genomes (six chromosomes) completely genome sequences (unpublished, personal communication) when publicly available will be used as reference for SNP call in our future research. Our current results in this article were good enough for the genetic diversity analysis of spinach using the SNP set and it is valuable to be published. This current version of our article has been the first one to examine genetic diversity in a USDA worldwide spinach germplasm collection with more than 200 accessions using a large set of molecular markers in spinach. Similar research for spinach genetic diversity was reported by Xu et al (2017) [16] among 120 cultivated and wild spinach accessions. However, only 51 spinach accessions were included from USDA GRIN. Among the 51 accessions, 25 accessions were crossed both reports in Xu et al (2017) and our current one. A total of 268 USDA germplasm accessions were included in our study with more spinach origin regions.

### Genetic diversity by geography–region and country

Spinach genetic diversity was associated with geographic origin–region and country, but there were some exceptions. Based on the phylogenetic analysis of the three regions, ‘Asia’, ‘Europe’, and ‘America’ in this study, the spinach lines from Europe were more similar to those from Asia than from America (Fig 5). The phylogenetic analysis based on the 12 countries where 6 or more spinach accessions were available, indicated that the materials from all five Asia countries (Afghanistan, China, India, Iran, and Japan) merged together into the same cluster; three Europe countries (Hungary, Macedonia, and Netherlands) merged together to form a single cluster; and the America material belonged to another cluster which was similar to the F1 hybrids and AR lines; but there was materials from Turkey and Belgium that represented exceptions and belonged to the Asia cluster (Fig 6).

Based on individual spinach lines, most of material from China, Japan, India, and Afghanistan merged together; most of Turkish lines grouped together; and most of lines from America merged together (S1 and S2 Figs). Wu et al. (2013) [13] also reported that there was a geographic effect existing in spinach genotypes based on 110 spinach germplasm accessions collected from different geographical origins in European, America, West Asia, East Asia, Northern China, Southern China and Japan and concluded that Northern and Southern Chinese spinach populations may have different origins from others. Xu et al (2017) [16] also reported there was geographic origin effect among the 120 spinach accessions tested and founded the cultivars from East Asia and Chinese commercial varieties cultivars different from those from Central/West Asia, Europe, North America and Africa. However, Hu et al. (2007) [11] indicated that the genetic relationships were not highly associated with the geographic locations based on 38 germplasm accessions and 10 commercial hybrids of spinach. The phylogenetic analysis based on 268 USDA GRIN spinach germplasm accessions in this study indicated that the spinach genetic diversity was associated with geographic origin–region and country.

### Special background existed in Arkansas spinach cultivars/lines

The spinach breeding program at the University of Arkansas has a long history, having been initiated over 40 years ago, and was one of the first public sector spinach breeding programs in the United States. The major breeding contributions have been in the areas of disease resistant cultivars, particularly to white rust, such as ‘Fallgreen’ released in 1987 [40] (Goode et al., 1988), ‘F380’ released in 1998 [5, 41] (Morelock et al. 2005, Morelock and Correll 2008), ‘Wintergreen’ released in 2003, and F415 [5] (Morelock et al. 2005). These are some of the most highly resistant genotypes to white rust, and have been widely used as germplasm for white rust resistance and as inbred parents by seed companies to develop resistant hybrids.

All 30 Arkansas lines merged together in the phylogenetic trees of all 343 spinach genotypes tested (S7 and S8 Figs) regardless there were two or four structured populations demarcated. Based on the phylogenetic analysis, the Arkansan lines F1 commercial hybrids were similar to the Arkansas lines. Based on region and country, the Arkansas lines were closer to America commercial F1 hybrids and then Netherlands F1 hybrids (Figs 5 and 6). From these results, we can concluded that the Arkansas lines had uniform and unique genetic background likely as a result of selection for white rust resistance. It also confirms that the Arkansas spinach breeding lines have been widely used as germplasm for white rust resistance and as inbred parents by seed companies to develop resistant hybrids. However, the selection process also has contributed to the Arkansas spinach lines having a relatively narrow genetic background. Thus it would be valuable for the Arkansas spinach breeding program to introduce spinach genotypes with different genetic background to develop broader genetic diversity and select for a wider range of desirable traits.

## Supporting information

S1 Table Spinach PI accession number, F1 hybrid, line, name, origin, country, region, seed source, cluster assigned in this study, and taxon name in 343 world-wide spinach genotypes. (XLSX)

S2 Table Spinach PI accession number, origin, country, region, and cluster assigned in this study in 268 USDA GRIN spinach accessions. (XLSX)

S3 Table Spinach F1 hybrid, origin country, region, seed source, and cluster assigned in this study in 45 F1 commercial hybrids. (XLSX)

S4 Table Spinach source, country, and the number of lines in each country. (XLSX)

S1 Fig The traditional phylogenetic tree combining structure populations (Q1 to Q2) from STRCTURE 2 and the Maximum Likelihood (ML) method from MEGA 6. The spinach accession number, the accession original country and region, and the structure population (cluster) were merged together into one taxon name as the each spinach accession ID in the drawn the combined tree draw by MEGA 6. The colored shape and branch are instead of one cluster matched the structure population (red round shape for Q1, green squared for Q2, and the black square with the black branch for the admixture Q1Q2 in 268 USDA GRIN spinach germplasm accessions.
(XLSX)

S2 Fig The traditional phylogenetic tree combining structure populations (Q1 to Q5) from STRCTURE 2 and the Maximum Likelihood (ML) method from MEGA 6. The spinach accession number, the accession original country and region, and the structure population (cluster) were merged together into one taxon name as the each spinach accession ID in the drawn the combined tree draw by MEGA 6. The colored shape and branch are instead of one cluster matched the structure population (red round shape for Q1, green squared for Q2, blue triangle for Q3, yellow diamond for Q4, purple triangle for Q5, and the black square with the black branch for the admixture in 268 USDA GRIN spinach germplasm accessions.
(XLSX)

S3 Fig The traditional phylogenetic tree combining structure populations (Q1 to Q2) from STRCTURE 2 and the Maximum Likelihood (ML) method from MEGA 6. The spinach F1 hybrid name, the accession original country and region, seed source company name, and the structure population (cluster) were merged together into one taxon name as the each spinach accession ID in the drawn the combined tree draw by MEGA 6. The colored shape and branch are instead of one cluster matched the structure population (red round shape for Q1, green squared for Q2, and the black square with the black branch for the admixture Q1Q2 in 45 F1 hybrids.
(XLSX)

S4 Fig The traditional phylogenetic tree combining structure populations (Q1 to Q3) from STRCTURE 2 and the Maximum Likelihood (ML) method from MEGA 6. The spinach F1 hybrid name, the accession original country and region, seed source company name, and the structure population (cluster) were merged together into one taxon name as the each spinach accession ID in the drawn the combined tree draw by MEGA 6. The colored shape and branch are instead of one cluster matched the structure population (red round shape for Q1, green squared for Q2, blue triangle for Q3, and the black square with the black branch for the admixture in 45 F1 hybrids.
(XLSX)

S5 Fig The traditional phylogenetic tree created by the Maximum Likelihood (ML) method from MEGA 6 in 91 spinach genotypes related to spinach sources from America. (XLSX)

S6 Fig The traditional phylogenetic tree created by the Maximum Likelihood (ML) method from MEGA 6 in 30 Arkansas spinach lines. (XLSX)

S7 Fig The traditional phylogenetic tree combining structure populations (Q1 to Q2) from STRCTURE 2 and the Maximum Likelihood (ML) method from MEGA 6. The spinach accession number/F1/line, the accession original country and region, and the structure population (cluster) were merged together into one taxon name as the each spinach accession ID in the drawn the combined tree draw by MEGA 6. The colored shape and branch are instead of one cluster matched the structure population (red round shape for Q1, green squared for Q2, and the black square with the black branch for the admixture Q1Q2 in 343 world-wide spinach genotypes.
(XLSX)

S8 Fig The traditional phylogenetic tree combining structure populations (Q1 to Q4) from STRCTURE 2 and the Maximum Likelihood (ML) method from MEGA 6. The spinach accession number/F1/line, the accession original country and region, and the structure population (cluster) were merged together into one taxon name as the each spinach accession ID in the drawn the combined tree draw by MEGA 6. The colored shape and branch are instead of one cluster matched the structure population (red round shape for Q1, green squared for Q2, blue triangle for Q3, yellow diamond for Q4, and the black square with the black branch for the admixture in 343 world-wide spinach genotypes.
(XLSX)
